# Thoracic Surgery Consultations in COVID-19 Critically Ill Patients: Beyond Conservative Approach

**DOI:** 10.1155/2021/6626150

**Published:** 2021-03-27

**Authors:** Yasser Aljehani, Sharifah A. Othman, Yousif Almubarak, Ayman Elbaz, Mohammed Sabry, Farouk Alreshaid, Hatem Y. Elbawab, Zeead M. Alghamdi, Mohammed Alshahrani

**Affiliations:** ^1^Division of Thoracic Surgery, Department of Surgery, King Fahad Hospital of the University, Khobar, Saudi Arabia; ^2^College of Medicine, Imam Abdulrahman Bin Faisal University, Dammam, Saudi Arabia; ^3^Department of Critical Care, King Fahad Hospital of the University, College of Medicine, Imam Abdulrahman Bin Faisal University, Dammam, Saudi Arabia; ^4^Faculty of Medicine, Menoufia University, Shibin Al Kawm, Egypt

## Abstract

**Introduction:**

Iatrogenic pneumothoracis, barotraumas, and tracheoesophageal fistulae, especially after prolonged intubation, and tracheal stenosis are all entities involving thoracic surgeons' consultation and management. With the surge of COVID-19 cases particularly in the critical care settings, various types of complications have been observed that require intervention from thoracic surgeons.

**Methods and Materials:**

A retrospective study was conducted in an academic healthcare institute in the Eastern Province of Saudi Arabia. We included all COVID-19 cases admitted to ICU in the period between March 15, 2020, and August 15, 2020, requiring thoracic surgery consultation and management. Non-COVID-19 critical cases and iatrogenic pneumothorax were excluded.

**Results:**

Of 122 patients who were admitted to ICU with COVID-19, 18 patients (14.75%) required thoracic surgery consultation and management. We discovered a significant association between the outcomes and reintubation rates and the rate of pneumothorax occurrence. The survival analysis showed improvement in patients who had thoracostomy tube insertion as a management than the group who were treated conservatively. On the other hand, there was a significant difference between the COVID ICU group who had thoracic complication and those who did not regarding the length of hospital stay.

**Conclusion:**

Noniatrogenic pneumothorax, subcutaneous emphysema, and mediastinal emphysema are well-known thoracic entities, but their presence in the context of COVID-19 disease is a harbinger for worse prognosis and outcomes. The presence of pneumothorax may be associated with better prognosis and outcome compared to surgical and mediastinal emphysema.

## 1. Introduction

The thoracic surgery role in critical care setting is usually reserved for complications. These could be related to a specific disease process, infections, inflammations, neoplasms, or other entities. They could also result from interventions performed within the intensive care unit (ICU).

Iatrogenic pneumothorax, barotraumas, and tracheoesophageal fistulae associated with prolonged intubations, and tracheal stenosis are all conditions involving thoracic surgery services in their management [1]. With the surge of COVID-19 cases particularly in the critical care setting, different types of complications are now requiring thoracic surgery consultation and management. Examples include subcutaneous emphysema (SE), mediastinal emphysema (ME), and noniatrogenic pneumothorax occurring individually or in combination [1].

The literature demonstrates that the significance of those entities is proportionate to the patient's prognosis, length of stay, and required recovery time. The pathophysiology indicates that the disease pattern rather than the side effects of assisted ventilation is the main cause of complications, with varying results depending on the lung area most affected by the disease [2].

We aimed to review and analyze COVID-19 critically ill patients requiring thoracic surgery consultation and management while in the ICU. Demography, patterns, predisposing factors, comorbidities, hospital stay, thoracic complications, lengths of stay, interventions, and outcomes were examined.

## 2. Materials and Methods

This retrospective chart review study was conducted in an academic healthcare institute located in the Eastern Province of Saudi Arabia. The inclusion criteria comprised all COVID-19 cases admitted to the ICU in the period from March 15th to August 15th, requiring thoracic surgery consultation and management. Non-COVID-19 critically ill patients and cases of iatrogenic pneumothorax were excluded from this study.

Patients' charts were reviewed, and demographic data, comorbidities, patterns of thoracic complications, management, length of stay, intubation, reintubation, ventilatory settings, intervention, outcome, and recovery were all analyzed. In cases of ME and SE, conservative management that included close follow-up clinically and radiologically was applied according to the clinical pathway for SE and ME [3]. Thoracostomy tubes were inserted in patients with pneumothorax that was discovered in the routine daily chest X-ray or in a chest X-ray that was performed whenever there is a clinical suspicion of pneumothorax occurrence.

Our study posed no physical, psychological, social, legal, or economic risks. The study protocol was approved by the Institutional Review Board (IRB) of Imam Abdulrahman Bin Faisal University, Dammam, Saudi Arabia.

### 2.1. Statistical Analysis

An excel spreadsheet was established for the entry of data. Data analysis was carried out using SPSS, Version 22. Categorical variables were summarized as frequencies and percentages. To determine the significant association of variables, the Mann–Whitney *U* test and Fisher exact test were used, and a significant level was considered at *P* < 0.05.

In addition, multivariate correlation and regression were done. Moreover, the Kaplan–Meier test was done for analyzing the expected duration of time of death in our sample size.

Finally, a comparison between our sample and the total number of COVID-19 patients who were admitted to the ICU regarding their outcome and total length of stay in the hospital was done by using the Kruskal–Wallis test.

## 3. Results

One hundred and twenty-two COVID-19 patients were admitted to the ICU between the period March 15, 2020, and July 15, 2020. Ninety-six patients (78.7%) were males and 26 (21.30%) were females, with a male : female ratio of 1 : 0.3.

Eighteen (14.75%) patients required thoracic consultation and management; fifteen males and three females (1 : 0.2) have developed thoracic complications including pneumothorax, SE, and ME. Fourteen patients required thoracostomy tube insertion. A descriptive analysis of our patients' population is shown in Tables 1 and 2.

The most common presenting symptoms were shortness of breath in 16 patients (88.9%), fever in 12 patients (66.6%), and cough in 11 patients (61.1%). Two patients (11.1%) were asymptomatic, one was admitted following a motor vehicle accident (MVA) and the second following a syncopal episode. One presented with hemoptysis and one with anorexia as an associated symptom.

During the hospital course, the mean of length of stay in the regular unit was 4.55 ± 26.94 days (ranging from zero to 21 days) and the mean of ICU length of stay was 24.11 ± 14.22 days (ranging from 6 to 52 days).

Most of the patients were on a maximum ventilation setting, and the mean duration of ventilatory support was 21.00 ± 14.9 days (ranging from 3 to 52 days). Two patients (11.1%) required reintubation, ventilatory setting programmed on pressure-regulated volume control (PRVC), with a median positive end-expiratory pressure (PEEP) of 14 ± 2.40 cmH_2_O (ranging from 10 to 18) and median FiO_2_ of 70% ± 19.64 (ranging from 40 to 100%). On average, the thoracic complications occurred on day 6 (±8.34 days) after intubation (ranging from 1 to 28 days).

**Figure 1:**
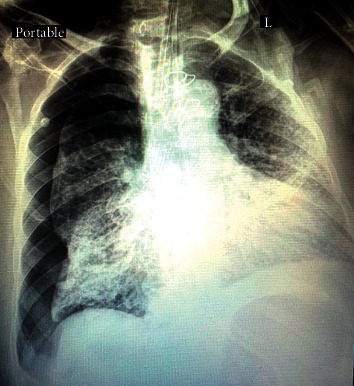
Pneumothorax with multiple scattered bilateral lung field air-pace opacities and diffuse ground-glass opacities.

**Figure 2:**
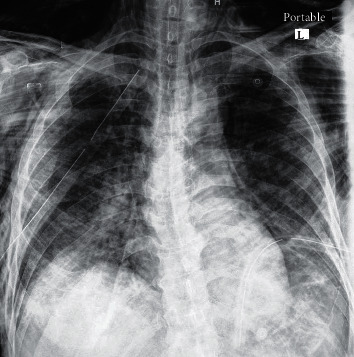
Chest X-ray showing pneumomediastinum and subcutaneous emphysema with inserted thoracostomy tube.

Four patients (22.2%) developed pneumothorax (Figure 1), six patients (33.3%) presented with only SE, one patient (5.6%) with only ME, and one (5.6%) had ME associated with SE (Figure 2). Six patients (33.3%) had pneumothorax associated with SE. Four patients (22.2%) were treated conservatively, and fourteen patients (77.8%) were treated with indwelling thoracostomy tubes. The mean duration of the thoracostomy tube placement was 6.72 ± 7.01 days. One patient required reinsertion of the thoracostomy tube for SE reaccumulation following a tracheostomy insertion. In our group of patients, 12 patients (66%) died as a result of the disease progression and organ failure, 4 patients (22.2%) discharged in good condition, while 2 patients (11.1%) are still hospitalized with slight improvement during the manuscript preparation.

There was a significant association between reintubation and the occurrence of pneumothorax and the outcome with *P* values of 0.034 and 0.007, respectively. The patients who required reintubation and those who had pneumothorax tend to have better prognosis.

Furthermore, multivariate correlation (Table 3) revealed a significant association between the duration of thoracostomy tube and pneumothorax (*P* value 0.005), reintubation (*P* value 0.011), outcome (*P* value 0.021), and duration of ventilatory support (*P* value 0.001). Pneumothorax showed significant associations with duration of ventilatory support (*P* value 0.002) and outcome (*P* value 0.012). A significant association between the duration of ventilatory support and reintubation was also noticed (*P* value 0.036) and with outcome (*P* value 0.005).

**Figure 3:**
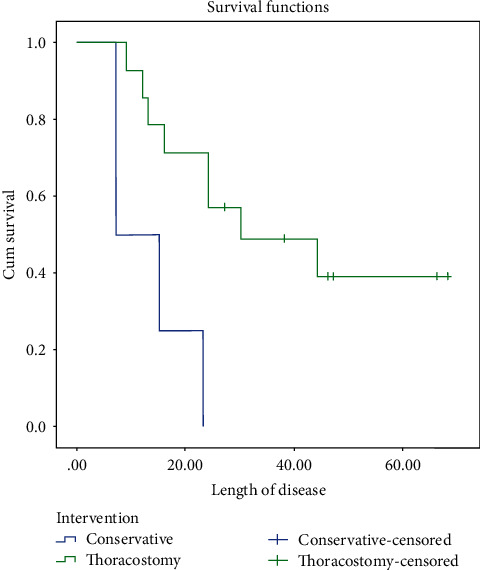
The survival curve of our patients between who underwent conservative treatment and thoracostomy tube insertion.

The Kaplan–Meier test was performed for analyzing the expected time of death in our sample depending on the intervention (i.e., conservative versus thoracostomy tube insertion). Insertion of the thoracostomy tube was associated with significantly better outcome and better survival rate (Table 4). The survival curve showed more favorable outcome for patients who had thoracostomy tube insertion in comparison with the conservative group (Figure 3).

In the whole COVID-19 ICU population, the occurrence of thoracic complications increased the length of hospital stay significantly (*P* value 0.026). However, it did not have an effect on the outcome (Table 5).

## 4. Discussion

In December 2019, the first case of COVID-19 was diagnosed in China. Human-to-human transmission transpired early in 2020, causing a global pandemic to ensue [4]. Since then, targeted medical strategies have been implemented at multiple levels encompassing numerous specialties. As the disease progressed, we developed an understanding of the disease components. We drew on experience gained earlier in the millennium from severe acute respiratory syndrome (SARS) and the influenza A virus subtype, H1N1 flu [4, 5]. The symptoms are similar to large extent, with a cough and shortness of breath as the hallmarks. Many physicians attribute coughing and the subsequent increase in intra-alveolar pressure as contributing causes of thoracic complications in such patients. In SARS-CoV-1, ME was typically seen in combination with SE or pneumothorax, rather than as solo entities [5]. It was also seen in unintubated patients, which supports the notion that the destruction of alveoli is due to the disease process rather than a complication of mechanical ventilation. The presence of ME is actually considered a marker for an increased likelihood of intubation [1]. Moreover, it was often a precursor of increased mortality. In SARS-CoV-1, ME incidence was around 11.6%. It is usually indicated by Naclerio's V sign, an air rim shadow on the border of the heart [1]. Unlike MERS-CoV and SARS-CoV-1, COVID-19 has a characteristic disease pattern of ground-glass opacities preferentially distributed towards the posterior segments of the lower lobes [6].

**Figure 4:**
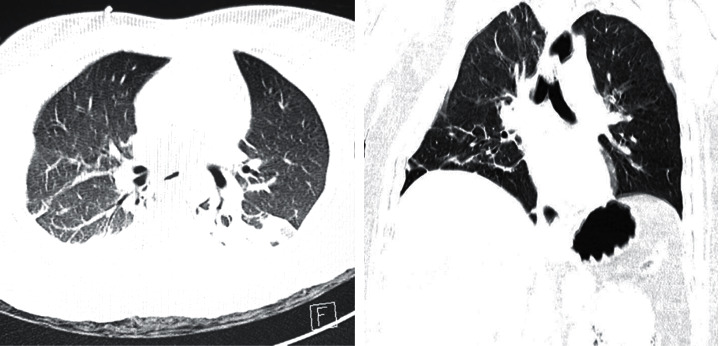
CT scan of COVID-19-positive patients, showing bilateral, subpleural, and peripheral ground-glass opacities, crazy paving appearance, and inter/intralobular septal thickening with air space consolidation.

Both diagnostic and therapeutic challenges exist when multiple nonspecific radiological signs present simultaneously. A subset of patients experiencing a unique set of complications have been noted during this pandemic. In terms of viral pneumonias, expected complications are due either to the disease itself, such as complicated pleural effusion, emphyema, and pneumothorax, or related to a particular therapeutic intervention provided. These can include iatrogenic pneumothorax secondary to the central line insertion or barotrauma from high-pressure ventilation support [6]. As SARS-CoV-2 reaches the airway, it advances until it reaches the alveoli. Replication of the virus then begins and causes exudation within the alveolar and interstitial spaces. This process is evident radiologically as ground-glass opacities seen in chest computed tomography scan (Figure 4) [7]. Lymphocytic infiltration leads to the destruction of the alveolar wall [8]. Air then leaks through the bronchovascular sheath and dissects through the mediastinum, causing ME, or advances distally, leading to SE [9]. Pneumothorax can result from the same process if located toward the periphery, or if the mediastinum leaks into the pleural space as a result of ballooning and overstretching [10]. The presence of these complications is a marker for disease progression, worse prognosis, and increased mortality [2, 11] by triggering respiratory failure and causing a vicious cycle in management [12].

In our study, we found a significant association between the outcome, reintubation rates, and these thoracic complications. In COVID-19 patients who developed thoracic complications in general, better prognosis was seen in patients with pneumothorax who were treated with thoracostomy tube insertion. The survival analysis test showed a higher survival rate in patients who were treated with indwelling thoracostomy tubes in comparison to the conservative group. The notion was that, in patients (other than COVID-19 patients) who developed SE and ME without pneumothorax, the conservative approach is well accepted [3]. But, we elected to treat by thoracostomy tube insertion on the ipsilateral side of the SE in our group of COVID-19 patients. The peculiarity of those patients and the isolation constraints in critical care setting and monitoring dictated to practice safer and more aggressive prophylactic approach.

A significant difference was observed between the total COVID-19 patients who were admitted in the ICU without development of thoracic complications and those who had thoracic complications in the total length of hospital stay, a reflection of the magnitude of these complications and their effect on patients.

It seems that the disease process plays a strong role in the degree of lung parenchyma destruction and subsequent thoracic complications. The generalization of such complications is the result of barotrauma which is not accurate since the first SARS infection. It is a true marker for aggressive disease that required this index of suspicion and prompt intervention.

## 5. Limitations

Due to the retrospective nature of this study, small sample size, and the fact that there is no consensus of clinical pathway in the literatures for COVID-19 patients, further studies involving multiple centers and a greater number of patients are required to clarify more statistical benefits and associations.

## 6. Conclusion

The COVID-19 pandemic has provided the medical world with multiple challenges. In terms of thoracic surgery, the occurrence of unusual thoracic entities and complications dictates the need to revisit some concepts. Noniatrogenic pneumothorax, subcutaneous emphysema, and mediastinal emphysema are well-known conditions, but their presence in the context of COVID-19 disease is a precursor for a dismal prognosis and outcome. The presence of pneumothorax may be associated with better prognosis and outcome compared to surgical and mediastinal emphysema. This would alert the critical care teams to have high clinical suspicion and to anticipate such complications.

## Figures and Tables

**Table 1:** Descriptive analysis of patients demographic data, hospital course, thoracic complications, intervention, and outcome.

General characteristics (No. = 18)	
*Age*	
Mean ± SD (range)	54.94 ± 13.07 (34–78)

*Gender*	
Male (%)	15 (83.3%)
Female (%)	3 (16.7%)

*Comorbidity*	
DM (%)	6 (33.3%)
HTN (%)	8 (44.4%)
DLP (%)	5 (27.8%)
CKD (%)	1 (5.6%)
Heart disease (%)	2 (11.1%)

*Presenting symptoms*	
SOB (%)	16 (88.9%)
Cough (%)	11 (61.1%)
Fever (%)	12 (33.3%)
Asymptomatic (%)	2 (11.1%)
Hemoptysis (%)	1 (5.6%)
Loss of appetite (%)	1 (5.6%)

*Hospital course*	
Length of unit admission (days) (mean ± SD (range))	4.55 ± 26.94 (0–21)
Length of ICU admission (days) (mean ± SD (range))	24.11 ± 14.22 (6–52)

*Ventilation*	
Length of intubation (days) (mean ± SD (range))	21.00 ± 14.99 (3–52)
Reintubation	
Yes (%)	2 (11.1%)
No (%)	16 (88.9%)
PEEP (median ±SD (range))	14 ± 2.40 (10–18)
PRVC (median ±SD (range))	70 ± 19.64 (40–100)

*Thoracic complications*	
Onset of complication after intubation (days) (median ±SD (range))	6 ± 8.34 (1–28)
Type	
Pneumothorax (%)	4 (22.2%)
Mediastinum emphysema (%)	1 (5.6%)
Subcutaneous emphysema (%)	6 (33.3%)
Pneumothorax and SE (%)	6 (33.3%)
SE and ME (%)	1 (5.6%)

*Intervention*	
Conservative (%)	4 (22.2%)
Thoracostomy (%)	14 (77.8%)
Reinsertion of thoracostomy	
Yes (%)	1 (5.6%)
No (%)	17 (94.4%)
Duration of chest tube (days) (mean ± SD (range))	6.72 ± 7.01 (0–20)

*Outcome*	
Alive (%)	4 (22.2%)
Died (%)	12 (66.7%)
Still admitted and slightly improved (%)	2 (11.1%)

ICU: intensive care unit; DM: diabetic; HTN: hypertension; DLP: dyslipidemia; CKD: chronic kidney disease; IHD: ischemic heart disease; CAD: coronary artery disease; SOB: shortness of breath; ME: mediastinum emphysema; SE: subcutaneous emphysema.

**Table 2:** Patients demographic data, hospital course, thoracic complications, intervention, and outcome.

#	Age	Gender	Comorbidity	Presenting symptoms	LOS unit (days)	LOS ICU (days)	Length of intubation (days)	Reintubation	Ventilator setting		Thoracic complication					Outcome
									PRVC	PEEP	Type	Onset from intubation	Intervention	Length of chest tube insertion	Reinsertion	
1	34	Male	Medically free	SOB, cough, fever	NA	7	3	No	50%	16	ME	1	Conservative	NA	No	Died
2	67	Male	Medically free	SOB	NA	24	22	No	70%	16	Pneumothorax	5	Thoracostomy	16	No	Died
3	78	Male	DM, HTN, CKD	SOB, fever	8	39	39	No	40%	14	Pneumothorax	1	Thoracostomy	8	No	Alive
4	37	Male	Medically free	Asymptomatic	NA	38	38	Yes	60%	14	Pneumothorax	3	Thoracostomy	16	No	Alive
5	70	Male	DM, HTN, CKD, IHD	SOB, cough, fever	16	52	52	No	70%	14	Pneumothorax and SE	11	Thoracostomy	13	No	Alive
6	45	Male	Medically free	SOB, cough, fever	3	21	3	No	40%	12	Pneumothorax and SE	20	Thoracostomy	1	No	Died
7	52	Male	HTN, DLP	SOB, cough	19	27	27	No	50%	10	Pneumothorax and SE	1	Thoracostomy	18	Yes	^*∗∗*^
8	66	Female	DM, HTN	SOB, cough, fever, loss of appetite	3	6	4	No	50%	10	SE	3	Thoracostomy	1	No	Died
9	65	Male	DM, HTN, DLP	SOB, cough, fever	21	45	45	Yes	70%	18	Pneumothorax and SE	28	Thoracostomy	20	No	Alive
10	52	Male	Medically free	SOB, fever	3	41	11	No	60%	12	SE	1	Thoracostomy	10	No	Died
11	67	Male	DM	Asymptomatic	6	17	17	No	100%	14	SE and ME	4	Conservative	NA	No	Died
12	39	Female	Medically free	SOB, cough fever, hemoptysis	NA	30	30	No	50%	14	Pneumothorax	25	Thoracostomy	5	No	Died
13	59	Male	Medically free	SOB	3	9	9	No	70%	12	SE	7	Thoracostomy	3	No	Died
14	40	Male	Medically free	SOB, fever	NA	7	7	No	70%	16	SE	7	Conservative	NA	No	Died
15	62	Male	CAD, HTN, DSL	SOB, fever	NA	16	16	No	100%	10	Pneumothorax and SE	10	Thoracostomy	6	No	Died
16	48	Male	DM, HTN	SOB, cough	NA	15	15	No	100%	12	SE	5	Conservative	NA	No	Died
17	45	Male	HTN, DSL	SOB, cough, fever	NA	27	27	No	70%	16	Pneumothorax and SE	16	Thoracostomy	3	No	^*∗∗*^
18	63	Female	DSL, hypothyroidism	SOB, cough, fever	NA	13	13	No	90%	16	SE	9	Thoracostomy	1	No	Died

ICU: intensive care unit; DM: diabetic; HTN: hypertension; DLP: dyslipidemia; CKD: chronic kidney disease; IHD: ischemic heart disease; CAD: coronary artery disease; SOB: shortness of breath; ME: mediastinum emphysema; SE: subcutaneous emphysema.  ^*∗∗*^Still admitted and slightly improved.

**Table 3:** Multivariate correlation of outcome, duration of ventilatory support, pneumothorax, reintubation, and duration of thoracostomy tube.

		Length of intubation	Reintubation	Pneumothorax	Length of tube	Outcome
Length of intubation	Pearson correlation	1				
	Sig. (2-tailed)					
	*N*	18				

Reintubation	Pearson correlation	−0.497 ^*∗*^	1			
	Sig. (2-tailed)	0.036				
	*N*	18	18			

Pneumothorax	Pearson correlation	0.683^*∗∗*^	−0.316	1		
	Sig. (2-tailed)	0.002	0.201			
	*N*	18	18	18		

Length of tube	Pearson correlation	0.714^*∗∗*^	−0.585 ^*∗*^	0.636^*∗∗*^	1	
	Sig. (2-tailed)	0.001	0.011	0.005		
	*N*	18	18	18	18	

Outcome	Pearson correlation	0.635^*∗∗*^	−0.287	0.580 ^*∗*^	0.538^*∗*^	1
	Sig. (2-tailed)	0.005	0.249	0.012	0.021	
	*N*	18	18	18	18	18

^*∗*^Correlation is significant at the 0.05 level (2-tailed). ^*∗∗*^Correlation is significant at the 0.01 level (2-tailed).

**Table 4:** Kaplan–Meier test for analysis of the survival rate in our sample regarding the management plan.

Means and medians for survival time								
Intervention	Mean^a^				Median			
	Estimate	Std. error	95% confidence interval		Estimate	Std. error	95% confidence interval	
			Lower bound	Upper bound			Lower bound	Upper bound
Conservative	13.000	3.830	5.494	20.506	7.000	—	—	—

Thoracostomy	40.404	6.541	27.584	53.224	30.000	15.173	0.261	59.739

Overall	34.314	5.800	22.947	45.682	24.000	1.054	21.934	26.066

^a^Estimation is limited to the largest survival time if it is censored.

**Table 5:** Comparison between group of COVID patients in the ICU who had thoracic complications and who did not, regarding length of hospital stay and prognosis.

Groups		*N*	Mean rank	*P* value
Length of hospital stay	With thoracic complications	18	78.58	0.026
	Without thoracic complications	104	58.54	

Outcome	With thoracic complications	18	58.83	0.680
	Without thoracic complications	104	61.96	

## Data Availability

The data used to support this study are available from the corresponding author upon request.
